# N-Myc and GCN5 Regulate Significantly Overlapping Transcriptional Programs in Neural Stem Cells

**DOI:** 10.1371/journal.pone.0039456

**Published:** 2012-06-26

**Authors:** Verónica Martínez-Cerdeño, Jessica M. Lemen, Vanessa Chan, Alice Wey, Wenchu Lin, Sharon R. Dent, Paul S. Knoepfler

**Affiliations:** 1 Department of Pathology and Laboratory Medicine, University of California Davis School of Medicine, Sacramento, California, United States of America; 2 Institute of Pediatric Regenerative Medicine, Shriners Hospital For Children Northern California, Sacramento, California, United States of America; 3 Department of Cell Biology and Human Anatomy, University of California Davis School of Medicine, Davis, California, United States of America; 4 University of Texas MD Anderson Cancer Center Houston, Texas, United States of America; Duke University Medical Center, United States of America

## Abstract

Here we examine the functions of the Myc cofactor and histone acetyltransferase, GCN5/KAT2A, in neural stem and precursor cells (NSC) using a conditional knockout approach driven by nestin-cre. Mice with *GCN5*-deficient NSC exhibit a 25% reduction in brain mass with a microcephaly phenotype similar to that observed in nestin-cre driven knockouts of c- or N-*myc*. In addition, the loss of *GCN5* inhibits precursor cell proliferation and reduces their populations *in vivo*, as does loss of N-*myc*. Gene expression analysis indicates that about one-sixth of genes whose expression is affected by loss of *GCN5* are also affected in the same manner by loss of N-*myc*. These findings strongly support the notion that GCN5 protein is a key N-Myc transcriptional cofactor in NSC, but are also consistent with recruitment of GCN5 by other transcription factors and the use by N-Myc of other histone acetyltransferases. Putative N-Myc/GCN5 coregulated transcriptional pathways include cell metabolism, cell cycle, chromatin, and neuron projection morphogenesis genes. *GCN5* is also required for maintenance of histone acetylation both at its putative specific target genes and at Myc targets. Thus, we have defined an important role for *GCN5* in NSC and provided evidence that GCN5 is an important Myc transcriptional cofactor *in vivo*.

## Introduction

*GCN5*/KAT2A was one of the first histone acetyltransferase (HAT) linked to transcriptional activation [1] and regulates a chromatin program of histone acetylation that is required for normal embryogenesis. *GCN5* null embryos exhibit elevated apoptosis in the mesodermal lineage [2]. More recently, mice with a conditional *GCN5* allele were created as well as mice with a knock-in of a catalytically dead *GCN5* (*GCN5* ^HAT/HAT^), which exhibit a neuronal phenotype with no apoptosis [3]. *GCN5* is also essential for normal embryonic stem cell pluripotency [4]. More recently loss of *GCN5* has been shown to accelerate cerebellar degeneration in a mouse model of neurodegeneration [5]. Despite these studies and the fact that *GCN5* is one of the most thoroughly studied HATs as part of the SAGA complex in yeast [1], very little is known about its function in normal development. Myc is the most well-studied DNA binding factor that recruits GCN5 to activate transcription, but E2F [6] and other factors such as p53 recruit it as well [7].

GCN5 also has a more global role in histone acetylation of unknown function [8] that could be involved in Myc’s global regulation of histone acetylation [9], [10]. Furthermore, the importance of *GCN5* for Myc function *in vivo*, such as during normal brain development, remains unknown as all studies of GCN5 function as a Myc cofactor have been conducted in immortalized cell lines**.** While *GCN5* substrates can include K9, K14 and K18 of histone H3 as well as all 4 amino-terminal K residues of histone H4 [11], the identity of endogenous substrates also remains an open question. We have previously found that the global chromatin function for Myc in maintaining AcK9 and acetylation of histone H4 in NSC appears to involve a role for *GCN5* as a Myc target gene [9]. However, the potential role of GCN5 protein as a cofactor for Myc in NSC *in vivo* remains unexamined in any developmental system.

The *myc* family of proto-oncogenes encodes chromatin regulatory proteins belonging to the basic-helix-loop-helix zipper (bHLHZ) class of transcription factors (reviewed in [12]). Related bHLHZ proteins called Mad/Mxd proteins antagonize Myc transcriptional functions and its influence on cell biology [13]. Both Myc and Mxd proteins bind DNA as dimers with a small, related bHLHZ protein, Max. Evidence for the antagonism between Myc and Mxd proteins on chromatin comes from the discovery that once bound to chromatin, Myc and Mxd proteins recruit opposite types of chromatin modifying enzymes. Beyond GCN5 [6], [14], Myc proteins recruit other HATs as well including TIP60/KAT5 [15] and p300/KAT3B, while Mxd proteins recruit histone deacetylases (HDACs) via the mSin3 corepressor [16], [17]. Chromatin immunoprecipitation assays (ChIP) have shown that Myc binding correlates strongly with histone acetylation, including acetylation of lysine 9 (AcK9) of histone H3 in the vicinity of specific E-box sites [18], while Mxd leads to deacetylation of the same residues [19]. Myc has been linked to histone lysine methylation as well via JARID demethylases [20]. This evidence along with the HAT and HDAC studies, points toward a central role for Myc and Mxd proteins in regulating the relative level of histone modifications via HDACs and HATs such as GCN5.

While excess *myc* is strongly associated with cancer, *myc* has also been linked with normal regulation of a variety of stem cells including NSC. It also plays an important role in the production of induced pluripotent stem cells (reviewed in [21]). Both c- and N-*myc* are constitutively required for embryogenesis [22], [23] as well as for embryonic stem cell function [24], [25], [26]. N-Myc plays a critical role in normal murine brain growth by controlling NSC function via regulation of global chromatin and specific target genes [9], [27], [28]. Nestin-Cre driven N-*myc* conditional KO in NSC severely disrupts murine brain growth, while c-*myc* disruption in NSC modestly impairs growth [29], [30]. The particular Nestin-Cre transgene used in these studies becomes weakly activated around E9.5 with apparent peak Cre expression in NSC *in vivo* around day E12.5 [31] and the transgene remains active in neurospheres as well [29]. A similar role for N-*myc* in human NSC is inferred from studies demonstrating that the human microcephaly syndrome, Feingold Syndrome, is caused by mutations in *MYCN* [32].

Normally, cortical precursor cells are located in two proliferative zones: the ventricular zone (VZ) and the subventricular zone (SVZ). VZ and SVZ precursor cells can be identified based on expression of the intermediate filaments nestin and vimentin [33], [34]. In previous studies, disruption of N- and/or c-*myc* affected precursors in both zones and attenuated cortical expansion [28]. Targeted gene disruption of either c-*myc* or N-*myc* or both in hematopoietic stem cells also alters their survival and self-renewal [35], [36]. A murine double KO (DKO) of both c-*myc* and N-*myc* using nestin-cre produced an extremely severe, often lethal phenotype including a near complete disruption of postnatal cerebellar development [27], [28].

The extent to which the *GCN5* gene or GCN5 protein are necessary for Myc’s chromatin, transcriptional, and biological functions *in vivo* during development is unknown. More specifically, the potential roles of Myc cofactors such as GCN5 in N-Myc function in NSC and brain growth remain important open questions. To address these issues, here we have used targeted gene disruption to knockout *GCN5* specifically in NSC both *in vivo* and *in vitro*. We found that *GCN5* is required in NSC for normal brain growth, producing a microcephaly phenotype similar to the nestin-cre driven *myc* KOs. In addition, loss of GCN5 decreases stem cell proliferation in the cerebral cortex. KO of *GCN5* in NSC produces gene expression changes that strongly overlap with those observed in N-*myc* KO NSC. Together these findings provide the first evidence that GCN5 and a Myc protein family member regulate significantly overlapping transcriptional programs *in vivo*.

## Results

### Production of Mice with Neural Stem and Precursor Cell (NSC) Specific Targeted Disruption of *GCN5*

**Figure 1:**
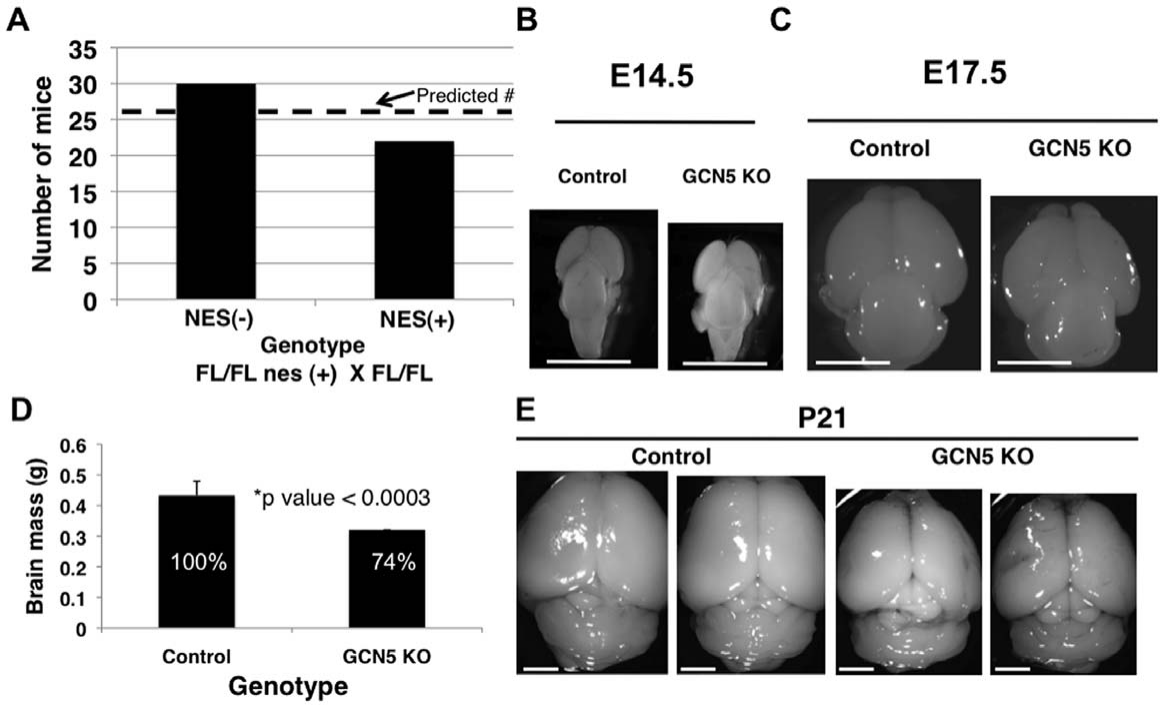
Mice with *GCN5* KO NSC have impaired brain growth. (A) Homozygously *GCN5* floxed mice were crossed with homozygously floxed nestin-cre+ mice, producing knockout mice (homozygous nestin-cre+) at a slightly less than predicted ratio. The dashed lines represent the number predicted based on Mendelian ratios. (B–C) Brains at E14.5 and E17.5 were isolated with evidence of impaired brain growth in the *GCN5* KOs at both stages. (D–E) Adult *GCN5* KO brains were smaller in mass and size. Scale bars are 2 mm in each image. Error bars are standard deviations.

To generate a *GCN5* KO specifically in NSC we took advantage of the well-characterized nestin-cre transgene, which we have previously used to make c- and N-*myc* NSC-specific KO mice [29], [30], [37]. We bred nestin-cre mice and mice with *GCN5* floxed alleles in which the vast majority of the *GCN5* coding region is flanked by LoxP sites [38]. We used a breeding strategy where we crossed *GCN5* flox/flox mice with *GCN5* flox/flox nestin-cre+ mice (Fig. 1A), producing 100% pups that are *GCN5* floxed. According to predictions from Mendelian genetics, in the absence of a lethal KO phenotype these pups should be 50% nestin-cre+ and 50% nestin-cre-. These crosses produced modest, but not statistically significant (p<0.08) evidence of lethality, as KO (nestin-cre+) mice were 15% underrepresented (Fig. 1A). Nonetheless, the surviving mice outwardly appear phenotypically normal with only a minor decrease in head size (not shown) that was suggestive of the microcephaly we have previously observed with nestin-cre mediated KO of c- or N-*myc*.

### Mice with *GCN5* Deficient NSC Exhibit Impaired Brain Growth and Microcephaly

To examine the overall effects of loss of *GCN5* in NSC on brain growth, we conducted timed matings, isolated embryos and microdissected E14.5 and E17.5 control flox/flox and *GCN5* KO (flox/flox nestin-cre+) brains (Fig. 1B–C). The size of the cerebral cortex and also the size of the cerebellum were proportionally reduced in the KOs. At E14.5 there was a visibly evident decrease in null brain size, particularly along the rostro-caudal axis (Fig. 1B). At E17.5, the *GCN5* KO brains were further reduced in relative size compared to littermate controls (Fig. 1C). Importantly, the size of the null cerebral cortex was decreased both in the rostro-caudal and medio-lateral axes at E17.5. We quantitated the reduction in size in different stages of the developing *GCN5* KO cortex. At E14.5, the null cortex was reduced 17% ±3 in the rostro-caudal axis, but it was not reduced in the medio-lateral axis. At E17.5, there was a reduction of 29% ±4 in rostro-caudal and of 25% ±4 in the medio-lateral axes. By adulthood the null brain was significantly reduced in mass (null mass was reduced to 74% of WT; Fig. 1D) and in size (Fig. 1E). In the *GCN5* null adult, the reduction in size was of 16% ±2 in rostro-caudal and 10% ±3 in the medio-lateral axes.

### Disruption of *GCN5* Results in Decrease in Precursor Cell Proliferation in the Developing Cerebral Cortex

**Figure 2:**
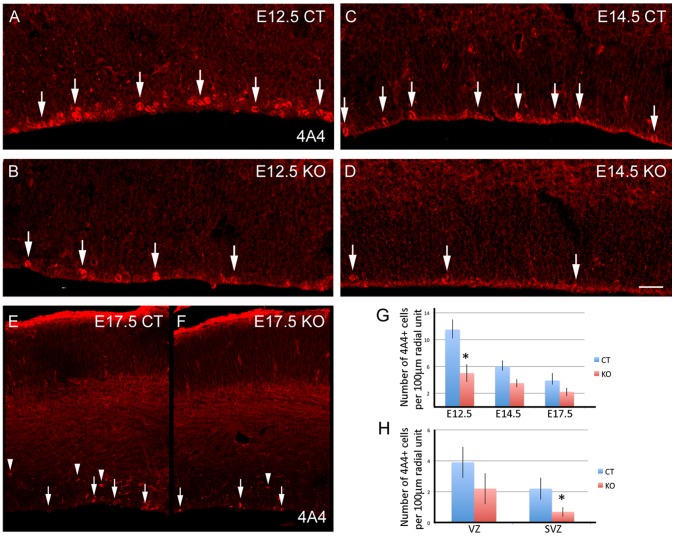
Loss of GCN5 reduces NSC proliferation and increases differentiation along the oligodendrocyte lineage. Proliferating 4A4+ cells in the ventricular zone (VZ) of E12.5 control (A), E12.5 *GCN5* KO (B), E14.5 control (C), and E14.5 *GCN5* KO (D). Notice than fewer cells are 4A4+ in the VZ of the KOs than in the control mice. Proliferating 4A4+ cells in the VZ and subventricular zone (SVZ) of E17.5 control and *GCN5* KO (E,F). There are fewer 4A4+ cells in the SVZ of the KOs than in the control mice. Some of the dividing 4A4+ cells at the VZ are indicated with an arrow, and the dividing 4A4+ cells at the SVZ are indicated with an arrowhead. Scale bar: A-F: 10 µm. Error bars are standard deviations.

The 26% reduction in brain mass in the adult *GCN5* NSC KO mice indicated that the loss of *GCN5* in NSC was inhibiting normal brain growth. Two potential causes of impaired brain growth are reduced cell cycling and increased apoptosis. To examine these possibilities we stained sections from the developing *GCN5* NSC KO mice with an antibody against phosphorylated vimentin (4A4), a marker for dividing precursor cells (Fig. 2). The *GCN5* KO ventricular zone (VZ) exhibited a marked decrease in the number of 4A4+ dividing precursor cells in all the developmental stages examined. We quantified the number of VZ 4A4+ cells present in a radial unit of dorsal cortex 100 µm wide spanning from the surface of the lateral ventricle to the pial surface. The number of 4A4+ cells in the null E12.5 VZ decreased by more than half (E12.5 control = 11.5±1.5 cells; E12.5 KO = 5.0±1.3, p = 0.01); the number of 4A4+ cells in the E14.5 VZ decreased almost by half, (E14.5 control = 6±0.9 cells, E14.5 KO = 3.5±0.6, p = 0.06); and the number of 4A4+ cells in the E17.5 VZ decreased by one third (E17.5 control 3.9±1.1, E17.5 KO = 2.2±0.6, p = 0.06; Fig. 2 A–G).

Effects of the E17. 5 *GCN5* KO were more pronounced specifically in the subventricular zone (SVZ) compared to the VZ. The number of 4A4+ mitotic cells in the SVZ was quantified in a radial unit of dorsal cortex 100 µm wide spanning from the surface of the lateral ventricle to the pial surface. We found that the number of SVZ mitoses in the E17.5 cortex was decreased three-fold in the *GCN5* KO (E17 control = 2.2±0.8, E17 KO = 0.7±0.4, p = 0.03, Fig. 2E–F, H). Together these data show that the cerebral cortex of the *GCN5* KO mice is reduced in size and that the number of dividing precursor cells per radial unit is decreased, pointing to a decrease in the total number of proliferating precursor cells in the *GCN5* null developing cerebral cortex. Since we did not observe a change in apoptosis by TUNEL staining in the *GCN5* KO (not shown), consistent with previous *GCN5* loss of function studies [3], our findings suggest that one major mechanism behind the decreased brain mass and size is reduced precursor cell proliferation.

### Separate Disruption of *GCN5* and of N-*myc* in NSC Leads to Strongly Overlapping Gene Expression Changes

**Figure 3:**
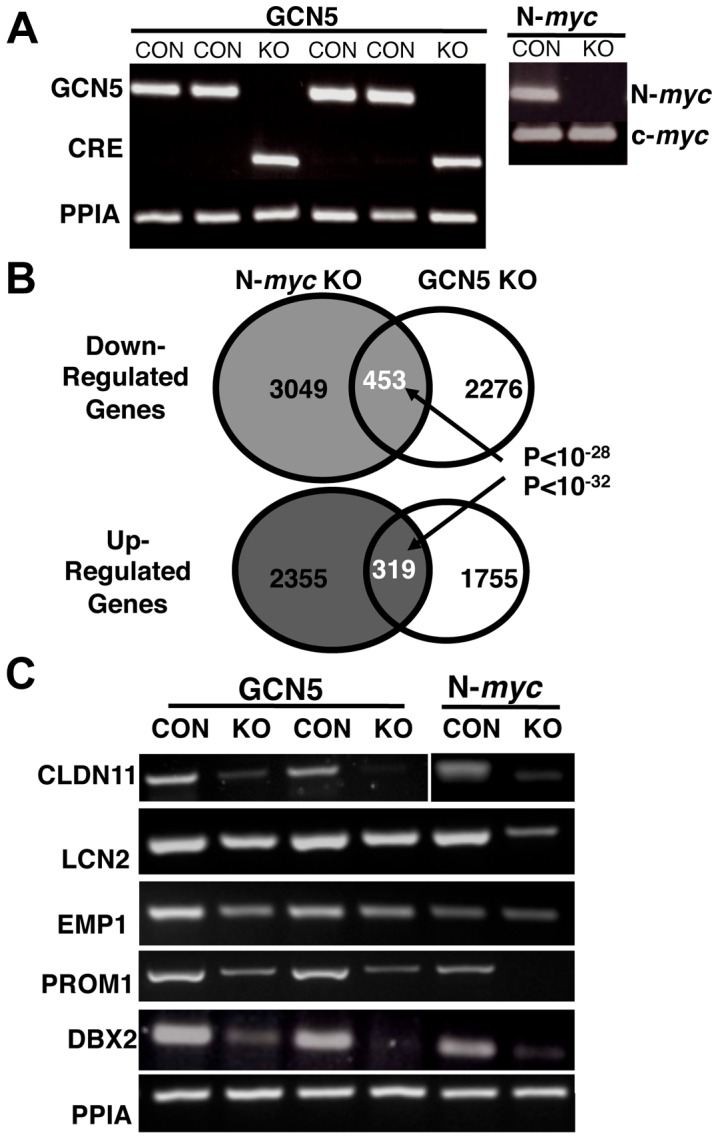
GCN5 and N-Myc regulate overlapping program of gene expression in NSC. (A) RNAs were isolated from control and *GCN5* KO neurosphere lines as well as from control and N-*myc* KO neurosphere lines. The KO in each case was verified by RT-PCR including PPIA for the loading control. (B) Expression microarrays were conducted on the RNAs and analyzed for overlap between genes altered in the *GCN5* and N-*myc* KOs. Overlap is indicated in the Venn diagrams along with p values. (C) Expression changes of select genes as measured by array were validated by RT-PCR.

To examine gene expression changes in the *GCN5* KOs, we established E12.5 neurosphere cultures from control (flox/flox) and *GCN5* KO (flox/flox nestin-cre) embryos. We isolated RNA from these neural stem and precursor cells and measured *GCN5*, Cre, and PPIA (loading control) levels by RT-PCR. As expected, we found that the *GCN5* KO neurospheres exhibited undetectable *GCN5* levels and N-*myc* null (N-*myc* floxed nestin-cre+) neurospheres did not express detectable N-*myc* (Fig. 3A).

Having validated the effectiveness of the KOs in neurospheres, the control and *GCN5* KO RNA samples were used for expression microarray studies, as were RNAs from control and N-*myc* KO neurospheres in parallel. To our knowledge, this was the first reported expression microarray study of a *myc* gene and a Myc protein cofactor gene, in this case *GCN5*, in parallel. The loss of N-*myc* resulted in the downregulation of approximately 4000 genes by 1.5 fold or more, while the loss of *GCN5* downregulated about 2700 genes (Fig. 3B). Strikingly, of the 2700 genes downregulated in the *GCN5* KO, 453 (17%) were also downregulated in the N-*myc* KO neurospheres (Table S1). This overlap in downregulated genes had a p value <10^−28^. There was also substantial (15%) overlap between upregulated genes in the *GCN5* and N-*myc* KO neurospheres, with a p value <10^−32^ (Table S2). Thus, a highly statistically significant fraction of genes was influenced by both N-Myc and GCN5 in the same manner in NSC in independent experiments.

### Ontological Analyses of Target Genes Indicate Roles for N-Myc and GCN5 in Regulating Stem and Precursor Cell Fate

Using DAVID gene ontological (GO) analysis (http://david.abcc.ncifcrf.gov), we analyzed the ontology of genes there were either down- or upregulated in N-*myc* and *GCN5* KO neurospheres (Tables 1–2). When analyzing the lists of actual genes with altered expression we focused further on genes with 2-fold or greater up or downregulation in both KOs (Tables 1–2). Of the downregulated genes that are putative targets of coactivation by N-Myc and GCN5, many exhibited very striking decreases in expression in both KOs (Table 1). A group with 10-fold or greater changes in both N-*myc* and *GCN5* KOs included the following: *CLDN11, LTK, H2-AB1, DSCR1L1 (RCAN2), SULF1, DBX2, GRIFIN, ITIH3, MYOM3,* and *EMP1. CLDN11* encodes OSP, Oligodendrocyte Specific Protein, which is specifically expressed in oligodendrocytes. The pronounced decrease in *CLDN11/OSP* expression in both KOs suggests that N-Myc and GCN5 are required for maintaining normal oligodendrocyte differentiation-associated gene expression. Many known Myc target genes were downregulated in the *GCN5* KO, including for example *EMP1/TMP* [39]. The genes most strongly upregulated by loss of either *GCN5* or N-*myc* included transcription factors such as *MITF*, which is important in retinal development and melanoma [40]. *EPHA5, ephrin A5*, was also strongly upregulated in both KOs and is important in axon guidance [40]. Importantly, the NSC marker gene, *Prom1* (CD133), was separately downregulated in both the N-*myc* and *GCN5* KOs. We chose six genes that exhibited downregulation in both KOs to analyze by RT-PCR, finding that the trends indicated in the array data were mostly confirmed, although changes in *LCN2* in the *GCN5* KO were at most mild (Fig. 3C). In addition, *EMP1* was not clearly downregulated in the *N-myc* KO neurospheres.

**Table 1:** Ontological Gene Clusters Upregulated in KOs (p values shown).

Category	N-*myc* KO	*GCN5* KO	SHARED GENES
**Phosphatase**	4.0×10^−23^	2.8×10^−12^	DUSP4, PTPRK, PTPRO, PTPRD
**Post-Synaptic Membrane**	2.2×10^−8^		
**Ras signaling**	1.0×10^−8^		
**Neuron differentiation**	1.7×10^−7^	3.2×10^−5^	BCL11B, ANK3, SLITRK1
**Peptidase (ADAM)**	1.6×10^−6^	7.9×10^−11^	ADAM12, ADAMTS5, ADAMTS1, ADAM23
**Synapse formation**	1.0×10^−6^	1.0×10^−6^	SYT1, SV2A, SYT9
**Condensed Chromatin**	1.3×10^−5^		

**Table 2:** Ontological Gene Clusters Downregulated in KOs (p values shown).

Category	N-*myc* KO	*GCN5* KO	SHARED GENES
**Ras signaling**	9.2×10^−17^	3.8×10^−13^	Diras1, RHOU, RAB3D
**Fatty acid metabolism**	1.6×10^−16^		
**Mitochondrial inner membrane**	1.1×10^−16^		
**Tight junctions**	1.0×10^−14^		
**Ubiquitin conjugation**	1.5×10^−10^	1.7×10^−17^	FBXO32, FBXO36
**Wnt signaling**	1.2×10^−9^	3.6×10^−5^	
**Peptidase Inhibitor**	1.6×10^−7^	1.0×10^−11^	ITIH3, many serpins
**EMP family**	1.0×10^−6^		
**Negative regulation of cell differentiation**	1.0×10^−6^		
**Notch signaling**	2.9×10^−5^		
**Negative regulation of neuron differentiation**	1.0×10^−5^		
**Cytochrome P450**		1.0×10^−13^	
**Cadherin**		2.2×10^−22^	
**DNA repair**		3.2×10^−11^	
**Neuron differentiation**		1.0×10^−7^	

Genes associated with neuronal differentiation and synapse formation were upregulated in both the N-*myc* and *GCN5* KOs, while genes involved in post-synaptic cell membrane function were up in the N-*myc* KO alone (Table 1). A set of genes known as neuron projection morphogenesis genes were upregulated in both the *GCN5* and N-*myc* KOs: *DSCAM, POU4F3, SLITRK1, APBB1, ANK4, EFNA5, GBX2, HOXA2, NGFR,* and *RELN*. These genes have a role in initiation, extension, and connection of axons and dendrites. For example, *DSCAM* mutant mice have defects in arborization of processes and spacing of cell bodies [41]. *SLITRK1* is expressed in mature neurons and its overexpression induces growth of unipolar neurites but not multipolar neurites [42]. *Gbx2*, expressed in the dorsal thalamus, controls growth and the capacity of neurites to reach their appropriate target [43]. In addition, *Bcl11a* is upregulated more than a 1000-fold in N-*myc* KO neurospheres and 4-fold in *GCN5* KO neurospheres. Bcl11a is a zinc-finger transcription repressor required to control axon branching and dendrite outgrowth [44]. More broadly, it is notable that independent knockout of N-*myc* or *GCN5* also led to downregulation of a host of the same transcription factor-encoding genes in addition to *Bcl11a* (Table 3).

**Table 3:** Transcription Factors Downregulated in Kos.

	Fold-changes	
	N-*myc* KO	*GCN5* KO
**BCL11a**	1739.0	4.0
**DBX2**	50.0	26.4
**HDAC11**	23.4	2.8
**HES5**	10.5	4.0
**JUNB**	8.2	4.4
**SHOX2**	5.1	20.9
**GATA2**	4.4	2.1
**PDLIM1**	2.9	3.2
**OTX1**	2.6	2.3
**TGIF**	2.4	3.6

### Evidence that N-*myc* and *GCN5* also Regulate Gene Expression in NSC Independently of Each Other

Both N-*myc* and *GCN5* have strong associations with ontological functional groups that are not shared between the two, suggesting some independent transcriptional functions (Tables 1–2). Interestingly, given our previous studies suggesting chromatin condensation in N-*myc* null NSC and a global role for Myc proteins in positive regulation of euchromatin, one class of gene upregulated in the N-*myc* KO (but not *GCN5* KO) was the “Condensed Chromatin” class. This might suggest that N-*myc* indirect regulation of chromatin through transcriptional control of effectors in NSC is at least partially GCN5-independent. N-*myc* alone also was positively linked to expression of genes involved in tight junctions, mitochondrial function, and fatty acid metabolism. *GCN5* was uniquely linked to positive expression of cytochrome P450, Cadherin, and DNA repair genes.

### GCN5 is Required for Maintenance of Histone Acetylation at N-Myc Target Genes and for more Widespread Histone Acetylation in NSC

**Figure 4:**
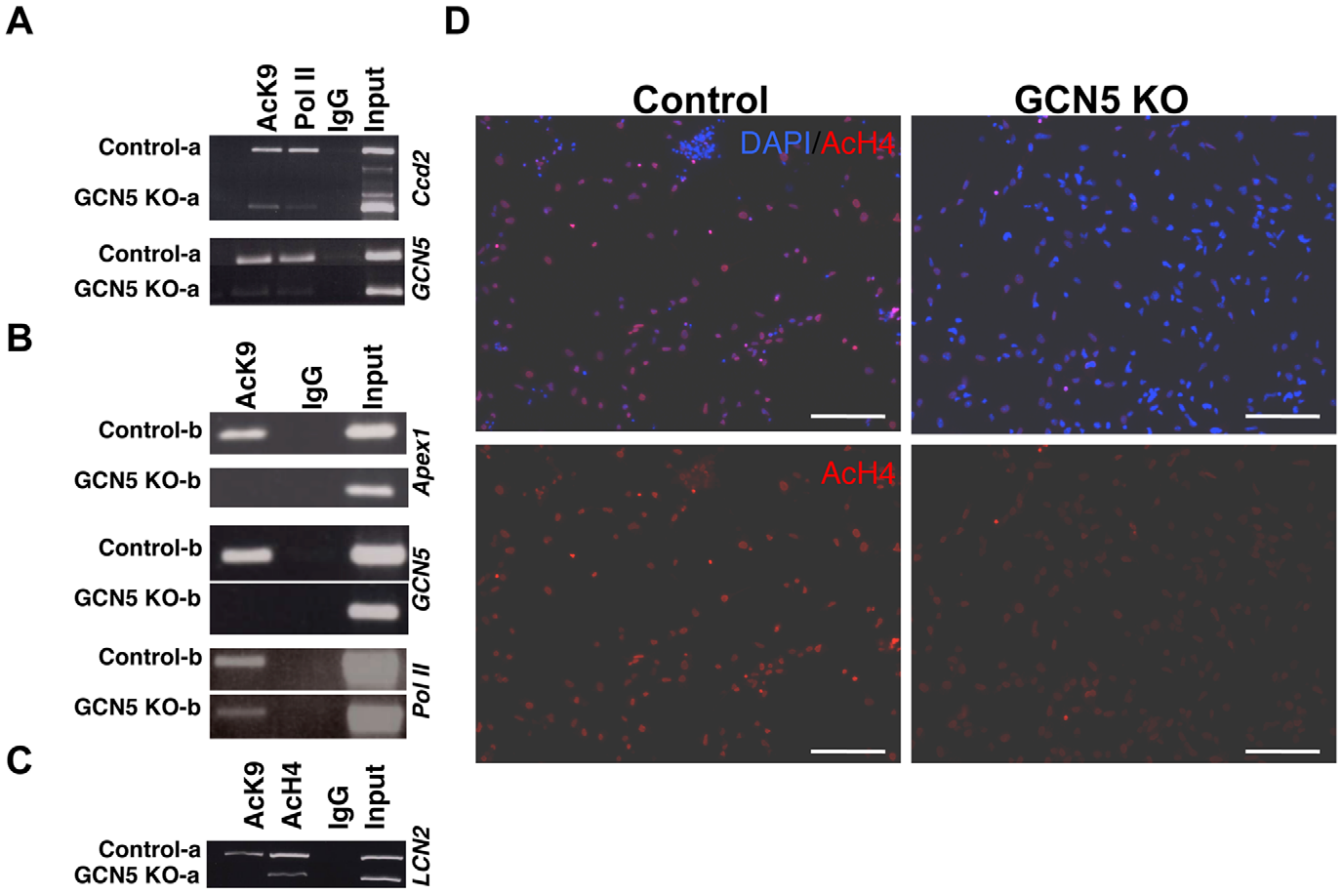
GCN5 maintains histone acetylation at specific target genes and in a widespread manner in NSC. (A–C) ChIP assays were conducted on control and *GCN5* KO NSC lines at both N-Myc (A–B) and *GCN5* target genes (C). *GCN5* binding itself was undetectable at any target. “a” and “b” represent biological replicate sets of neurospheres isolated from littermate embryos. (D) Control and *GCN5* KO NSC were immunostained for pan-Ac-H4 (Red) and DAPI (Blue). Scale Bars are 100 µM.

To explore the potential role of N-Myc recruitment of GCN5 in mediating histone acetylation at N-Myc target genes, we conducted ChIP assays. ChIP assays for murine GCN5 using four commercially available antibodies did not in any case produce a detectable band (not shown) at any of the N-Myc or GCN5 target genes. However, focusing on a key GCN5 target for acetylation (lysine 9 of histone H3; AcK9), we conducted ChIP assays for AcK9 as well as RNA Pol II at N-Myc target genes. We found that *GCN5* KO NSC had greatly decreased levels of both histone acetylation and RNA Pol II (Fig. 4A) at the *GCN5* and *ccd2* (*cyclin D2*) gene promoters. We found the same pattern using a second independent set of control and *GCN5* KO NSC (Fig. 4B), finding decreased AcK9 at the promoters of *Apex1*, *GCN5*, and *Pol II*. GCN5 appears to be required to maintain acetylation of both histone H3 (AcK9) and histone H4 as both AcK9 and AcH4 (pan-acetylated histone H4 antibody) were reduced at the *Lcn2* gene promoter in *GCN5* KOs (Fig. 4C). When we looked at histone acetylation levels more globally by immunostaining of NSC, we found no clear decrease in global AcK9 (not shown) in *GCN5* KOs, but a striking decrease in acetylation of histone H4 (Fig. 4D). These findings suggest GCN5 is key for the maintenance of AcK9 at specific target gene promoters and more widely for overall acetylated histone H4 levels.

## Discussion

This study is the first to measure in parallel the potential cooperative functions of a transcription factor (N-Myc in this case) and its recruited HAT cofactor (GCN5) using mouse genetics to perform parallel target gene disruption. We found that the HAT GCN5 is required in NSC for brain growth and produces a phenotype with both some similarities and differences from the N-*myc* KO phenotype using the same nestin-cre. Parallel expression microarray studies indicated that approximately 1/6 of genes regulated by GCN5 are also regulated by N-Myc. The simplest model to explain this overlap is that N-Myc is recruiting GCN5 to these genes to control their expression, a model that fits for genes that are downregulated by KO of N-*myc* or GCN5. However, interestingly there is also strong overlap between genes upregulated in both N-*myc* and *GCN5* KO NSC, representing putative targets of N-Myc and GCN5 repression. The striking overlap in genes activated by either N-Myc or GCN5 loss-of-function (representing genes that would apparently be repressed by N-Myc and GCN5 normally by some mechanism) suggests, not surprisingly, that at least some indirect effects on gene expression occur since histone acetylation driven by GCN5 is proposed to strictly activate genes. One possible model for derepression of genes in both knockouts is that N-Myc and GCN5 work together to activate expression of genes encoding repressor proteins that in turn suppress expression of genes in specific functional pathways.

Of downregulated genes, N-Myc and GCN5 null NSC shared many ontological clusters including, most prominently, those related to signaling including Ras and Wnt. If N-Myc and GCN5 function together, in part, to coordinately regulate Ras and Wnt signaling, then the disruptions in these pathways in the KOs may, at least in part, explain the changes in cell fate observed in both KOs. Importantly, while both N-*myc* and *GCN5* KOs exhibited a downregulated Wnt cluster, the genes within the clusters were entirely different as no specific Wnt pathway genes were downregulated in both KOs. This suggests that N-Myc and GCN5 have distinct effects on Wnt signaling. In addition, the Ras clusters downregulated in each KO only partially overlapped, indicating that GCN5 and N-Myc regulate both distinct and shared aspects of NSC signaling, likely explaining the differential effects of their loss on cell fate. Together these findings suggest that N-Myc and GCN5 may in part influence development via complex regulation of signaling pathways.

The strong downregulation of ubiquitin pathway genes (e.g. FBXO32 and FBXO36) in both KOs supports the model in which N-Myc and GCN5 together are positive regulators of the activity of the proteome, and suggests in their absence there are not only transcriptional changes but also widespread alterations in protein levels due to enhanced stability. These posttranscriptional events are also likely mechanisms underlying the brain growth and fate phenotypes observed in the KOs. N-Myc and GCN5 also both strongly inhibit phosphatase and peptidase gene expression (the expression of genes in these classes are upregulated in both KOs), while interestingly N-*myc* and *GCN5* are also required for maintained expression of peptidase inhibitors. These data strongly suggest N-*myc* and *GCN5* have a role in delimiting peptidase activity in NSC.

While the overlap between genes transcriptionally regulated by GCN5 and N-Myc was highly significant statistically, the presence of a majority of gene expression changes that were not shared also suggests a significant divergence of many functions. More specifically, it argues that N-Myc is frequently recruiting other HATs, such as TIP60 and p300, to regulate NSC functions, and that GCN5 is often recruited by transcription factors besides N-Myc. The dramatic cerebellar phenotype in the nestin-cre driven N-*myc* KO mice coupled with the absence of any disproportionate cerebellar phenotype in the nestin-cre driven *GCN5* KO mice, indicates that N-Myc protein likely functions to direct cerebellar development independently of GCN5 through another HAT. Thus, an intriguing question is whether *GCN5* has any role in the genesis of the cerebellar tumor medulloblastoma that is frequently associated with *Myc* family gene amplification. Future studies knocking out additional Myc cofactors such as TIP60, p300, and JARID factors in NSC employing the same nestin-cre are likely to shed further light on Myc function in brain development and brain tumors. In addition KOs of other transcription factors that recruit *GCN5* using nestin-cre may provide mechanistic insight into not only their function, but also that of *GCN5*.

The enhanced neuronal differentiation phenotype in the N-*myc* KO *in vivo* and in NSC is reflected in the changes in the gene expression array studies, including upregulation of neural differentiation genes by ontological analysis and downregulation of a cluster of differentiation inhibitory genes. *GCN5* KO also results in upregulation of some neural differentiation genes, including some that are shared with N-Myc such as BCL11A, BCL11B, ANK3, and SLITRK1. Interestingly, *GCN5* KO leads to downregulation of neuronal differentiation genes, the gene expression changes that are perhaps dominant in the *GCN5* KO given the impaired neuronal differentiation observed *in vivo* in the *GCN5* KO.

Our studies of the parallel loss-of-function between N-Myc and GCN5, supporting the model of their cooperative function to regulate gene transcription and NSC biology, suggest that other parallel transcription factor/histone modifying enzyme cofactor knockout studies incorporating both phenotypic analysis and gene expression analysis may prove fruitful. The findings that N-Myc and GCN5 appear to work together to maintain a progenitor-like state of inhibited differentiation, high cellular metabolism, and rapid proliferation also support a model in which GCN5 may mediate aspects of nervous system tumor formation by excess N-Myc when such a progenitor-like state appears to be “locked in.” Further studies will provide additional insight into the functions of N-Myc and GCN5 in NSC and tumorigenesis.

## Methods

### Immunohistochemistry and Measurements

All E12.5 embryos were immersion-fixed overnight in fresh 4% paraformaldehyde (PFA) in Phosphate Buffered Saline (0.1 M PBS), paraffin embedded and 12 µm sagittal slices were cut in a microtome. Pregnant females were anesthetized with 150 mg/kg ketamine and 16 mg/kg xylazine, then E14.5 and E17.5 embryos were removed individually and perfused with PFA. Brains were removed and fixed overnight in fresh PFA, then cryopreserved with 30% sucrose and 12 µm coronal slices were cut in a cryostat. For immunostaining, E12.5 embryo sections were deparaffinized then rehydrated and 30 mM sodium citrate treated for 10 min at 95°C, while E14.5 and E17.5 frozen brain sections were post fixed at −20°C acetone for 10 min followed by three 5-minute PBS washes. All sections were blocked in 10% normal goat serum in PBS for 1 hr at room temperature then incubated in primary antibody overnight at 4°C. Antibodies used include anti-phosphorylated vimentin (Mouse anti-4A4,1∶1000, MBL, D076-3, Toronto, Canada), Goat anti-Olig2 (1∶250, R&D Systems, AF2418, MN, USA), Mouse anti-NeuN (1∶1000, Chemicon, MAB 377, MA, USA), Rabbit anti-S100 (1∶500, Abcam, ab52642, Cambridge, UK). The next day sections were washed in PBS three times for 10 min each, and then incubated in secondary antibody for 2 hrs at room temperature. Donkey anti-Rabbit, donkey anti-Goat, and donkey anti-Mouse were used (1∶200, Jackson Immunoresearch Laboratories, PA, USA). Sections were washed again in PBS three times for 10 minutes each, and then mounted in Vectashield mounting medium with DAPI (VECTOR H-1200, CA, USA). TUNEL staining was performed using the DeadEnd Fluorometric TUNEL System Kit (Promega G3250, WI, USA) as directed.

For quantitation of proliferation by 4A4 staining we used n = 3 for each group and the standard deviations are from different embryos. Changes in the sizes of brains were quantified by using microscopy and image analysis to measure sagittal (length) and coronal (width) slices.

### ChIP Assays

Chromatin samples were prepared as previously described [45]. Neurospheres were crosslinked as monolayers. Antibodies used for ChIPs include the following: N-Myc (2 µg Abcam 16898, Cambridge, UK), AcK9 (2.5 µg 06–942 Upstate, MA, USA), and AcH4 (Upstate 06–866, MA, USA). 2 µg Rabbit IgG was used as a background control. Immunoprecipitated chromatin fragments were amplified by PCR using primers specific for each promoter region. If inputs between *GCN5* control and KOs were substantially different, samples were re-run. Murine GCN5 ChIPs were unsuccessfully tried with the following antibodies: Abcam 18381, Cell Signaling 3305, Santa Cruz 20698, and Santa Cruz 6303. In those experiments IPs were conducted with antibodies for other factors including N-Myc, Pol II, and AcK9H3, which all worked indicating that the ChIP assays themselves worked, but that murine GCN5 is not ChIP’able using these antibodies.

ChIP primers.

mCcd2 prom 1: GGC TTA AGA AGC ACC CCT TT.

mCcd2 prom 2: CTC TAA CTT CGG AGG CTT GC.

m*GCN5* L2 1: AGC ATC CAC AAA TCC TCC AG.

m*GCN5* L2 2: TAG TCT TCC TTT GCC GCT TC.

mApex1 1: ACG AAC AAC CCA GAA CCA AG.

mApex1 2: CTA AGC CAG AGA CCC TCA CG.

mPolII 82s: GAC GGG TTC TGA GCA CTT AG.

mPolII 151a: CAA CAT CAG CAT CAC TGA CC.

Lcn2 1: GTG CTT AGC TCC TAT ATG CAG C.

Lcn2 2: CTG AGC AGG GCA TAG ATC CTT.

### Microarray and RT-PCR

AGE Total RNA was isolated from control and *GCN5* KO neurospheres (the same set of samples as shown in Fig. 3A) using RNeasy Mini Kit (Qiagen 74134, CA, USA) with DNaseI digestion (Invitrogen 18068–015, CA, USA) performed post RNA extraction after approximately 1 month in culture. Quality of RNA was checked and cRNA was produced by UC Davis Gene Expression Analysis facility for hybridization to Sentrix Mouse Ref-8 Expression microarray. RT-PCR was performed on total RNA from the same batch used for array. Four GCN5 control (CON) and two GCN5 knockout (KO) biological replicates were used for the array data analysis, and the genes indicated in Fig. 3B as up- or downregulated were restricted to those showing consistent changes in replicates.

### Neurosphere Assays

Neurospheres were isolated, cultured, and differentiated as previously described [29].

## Supporting Information

Table S1 **List of genes downregulated both in GCN5 KO NSC and in N-Myc KO NSC. Genes are listed by name and sorted in descending order by fold change in the GCN5 KO.**
(PDF)

Table S2 **List of genes upregulated both in GCN5 KO NSC and in N-Myc KO NSC. Genes are listed by name and sorted in descending order by fold change in the GCN5 KO.**
(PDF)
