# Supplementary material for: N-Myc and GCN5 Regulate Significantly Overlapping Transcriptional Programs in Neural Stem Cells
Source: PLoS One. 2012 Jun 26;7(6):e39456. doi: 10.1371/journal.pone.0039456 (PMC3383708; doi:10.1371/journal.pone.0039456)
Supplement: Table S1 — List of genes downregulated both in GCN5 KO NSC and in N-Myc KO NSC. Genes are listed by name and sorted in descending order by fold change in the GCN5 KO. (PDF) [file pone.0039456.s001.pdf]

Supplemental Table S1 Genes downregulated both in GCN5 and N-Myc KO NSC

TargetID	Expression Control/KO GCN5	Expression Control/KO N-Myc
CLDN11	84.2	64.9
SLC5A4A	65.4	3.5
LTK	54.2	10.2
OLFR295	54.2	2.2
TNFRSF5	40.7	5.4
H2-AB1	34.1	12.3
TRBV12-1	29.1	3.1
DSCR1L1	27.5	42.8
DEFCR-RS7	27.0	4.2
SULF1	26.4	30.4
DBX2	26.4	50.0
VAX2	22.2	2.6
PROM1	22.0	6.0
OLFR738	22.0	3.4
SHOX2	20.9	5.1
GRIFIN	20.2	10.2
SCL0326621.17	20.2	2.2
IGFBPL1	18.4	4.0
DNASE2A	15.9	3.4
MFAP4	14.8	5.9
ITIH3	14.6	158.2
MYOM3	14.4	22.8
COL9A1	13.5	3.8
DDX4	13.1	4.7
AI450948	12.4	2.9
TM4SF11	11.4	4.3
STXBP2	11.2	6.5
EMP1	10.3	66.8
HOXA7	10.0	2.3
TLR5	9.9	38.8
ZC3HAV1	9.7	3.5
CCR4	9.4	3.4
TRPM8	8.9	2.1
MATN4	8.9	2.3
SLFN10	8.9	2.5
PCDHB9	8.7	50.2
I830077J02RIK	8.3	5.0
CYBRD1	8.2	4.8
ARPM1	7.4	2.0
STAC2	7.3	2.4
MKRN1-PS1	6.9	4.3
STK32A	6.7	8.2
FBLN2	6.4	2.0
IGHV3S2	6.4	2.0
ITGA2B	6.2	2.1
MEIG1	6.2	20.0
MUC1	6.1	5.4
RCN3	6.0	2.6
RGS16	5.9	45.5
PTGS1	5.7	2.1
PEX11C	5.7	2.0
OLFR116	5.6	17.6
PSMB8	5.6	2.6
CHST3	5.4	3.8
HTR3A	5.3	26.6
ABCC12	5.2	2.3
PIK3CD	5.2	14.2
GSTT1	5.1	29.4
OLFR1307	5.0	227.0
MOV10	4.9	4.5
AI790298	4.9	2.3
CAR14	4.8	7.2
TRPC2	4.7	2.1
IGHV2S4	4.7	3.6
PGBD5	4.6	4.4
LIPH	4.5	3.2
OCM	4.4	2.1
LCN2	4.4	61.9
FOXL2	4.4	3.2
IL3RA	4.4	2.3
JUNB	4.4	8.2
FV4	4.3	8.4
AI451557	4.3	4.4
PAM	4.3	270.6
ADCYAP1	4.3	2.5
RHCG	4.2	3.4
MYL9	4.2	162.9
AI450757	4.1	6.5
GADD45G	4.1	6.3
HES5	4.1	10.5
CSNK2B	4.1	2.4
HOOK2	4.1	8.8
BCL11A	4.1	1739.3
KLK15	4.0	2.0
GCAP14	4.0	2.6
SPATA13	4.0	6.4
NID1	4.0	2.6
STX11	3.9	7.9
TIMP1	3.9	30.4
GABABRBP	3.9	5.2
SCL000625.1_4	3.9	4.2
AB023957	3.9	15.7
IGFBP5	3.9	8.2
AI427122	3.9	20.2
ANXA9	3.9	2.8

Supplemental Table S1 Genes downregulated both in GCN5 and N-Myc KO NSC

DNCL2B	3.9	4.3
OCIL	3.8	9.1
SLC14A2	3.7	11.5
RHPN2	3.7	3.3
RNASE1	3.6	16.9
PLEKHA2	3.6	2.3
TGIF	3.6	2.4
MTDNA_ND6	3.6	2.1
LENEP	3.6	8.6
AKAP3	3.5	3.4
EVC2	3.5	2.3
CML4	3.4	145.0
FGL2	3.4	2.6
TLR2	3.4	2.3
MJD	3.4	2.5
IGF2BP3	3.4	12.7
MGLL	3.4	12.3
CD109	3.4	12.6
SPIRE2	3.4	5.5
LGALS9	3.3	3.4
MMP23	3.3	3.6
FLI1	3.3	3.9
AA175286	3.3	11.3
PLCD1	3.3	4.6
SLC7A4	3.3	17.0
MFNG	3.2	3.9
ACTG1	3.2	5.7
TULP2	3.2	3.6
PDLIM1	3.2	2.9
ADAMTS19	3.2	4.6
DHRS6	3.1	18.8
S100A13	3.1	4.9
GUCY1B2	3.1	14.0
AA409316	3.1	12.7
ZFP319	3.1	2.1
PRG2	3.1	2.1
HOXB7	3.1	2.4
MSX2	3.1	2.6
ENPP2	3.1	4.0
COL6A1	3.1	30.0
RAB5A	3.0	3.4
SPARC	3.0	8.3
RMCS1	3.0	18.0
SCL0002976.1_36	3.0	2.9
IRF5	3.0	2.6
V1RB4	2.9	2.7
CD9	2.9	3.5
OLFR139	2.9	2.1
HFE	2.9	4.0
POLYDOM	2.9	2.3
SLP	2.9	95.0
MEF2B	2.9	4.2
CEECAM1	2.9	2.5
FCRL3	2.9	5.4
PLCD3	2.9	3.5
PLSCR4	2.9	3.4
H2-T10	2.8	2.3
INSL6	2.8	3.1
SEC1	2.8	7.6
HDAC11	2.8	23.4
SCL000648.1_30	2.7	2.7
KDT1	2.7	6.0
ADFP	2.7	6.4
APOC4	2.7	8.4
CEP2	2.7	6.2
HEPH	2.7	8.2
LOC230872	2.7	7.7
DLEU2	2.6	2.4
GJA9	2.6	4.1
EPB4.1L4A	2.6	5.1
IGTP	2.5	20.7
CARD10	2.5	3.3
CAPON-PENDING	2.5	2.7
THBS3	2.5	3.8
MVD	2.5	2.2
PPAP2C	2.4	8.9
IGF1	2.4	12.2
ADAMTS4	2.4	5.6
FBXO36	2.4	4.1
COL6A2	2.4	2.6
SCL0002975.1_346	2.4	2.2
SCL0001356.1_58	2.4	2.5
ADAMTS15	2.4	3.3
ZFP352	2.4	4.5
CSH1	2.4	2.0
GNG11	2.4	2.7
RIBC1	2.4	2.2
FBXO32	2.4	6.0
RESP18	2.3	3.7
NOL3	2.3	3.8
ITGB8	2.3	5.1
CD24A	2.3	2.8
SOCS3	2.3	9.3
OPRL	2.3	4.5
BZRPL1	2.3	3.2
YM24D07	2.3	3.0
DLL1	2.3	2.8
PARD6A	2.3	2.7

Supplemental Table S1 Genes downregulated both in GCN5 and N-Myc KO NSC

SCL0002711.1_76	2.3	7.8
KHK	2.3	4.4
OTX1	2.3	2.6
OLFR624	2.3	2.5
RDH5	2.3	8.0
SCL000048.1_50	2.2	2.9
FHOD1	2.2	4.2
SHRM	2.2	4.6
CYP4V3	2.2	19.8
GPR133	2.2	3.7
BCL2L11	2.2	2.5
ELA1	2.2	3.7
FBXL8	2.2	4.0
RAB17	2.2	3.5
EPHA2	2.2	39.6
CSRP3	2.2	3.1
PCDH15	2.2	5.7
NTE	2.2	3.5
MAST1	2.2	2.7
SCL0004060.1_43	2.2	3.5
PADI2	2.2	23.2
OLFR100	2.2	7.1
LOC432611	2.1	3.5
DNAJC12	2.1	3.6
FETUB	2.1	6.4
LPXN	2.1	2.4
KCNJ10	2.1	5.9
EMP2	2.1	6.0
SPIB	2.1	3.9
RHOU	2.1	3.9
TNFRSF4	2.1	7.2
IRF1	2.1	2.5
MSRB	2.1	4.3
RAB3D	2.1	2.9
GATA2	2.1	4.4
DIRAS1	2.1	3.0
GPR30	2.1	3.6
IRX1	2.0	59.9
MCF2L	2.0	2.6
H2-L	2.0	2.4
SCL000723.1_11	2.0	3.6
MYD88	2.0	8.2
SCL0003943.1_25	2.0	3.7
MAP3K8	2.0	9.6
