# Supplementary material for: N-Myc and GCN5 Regulate Significantly Overlapping Transcriptional Programs in Neural Stem Cells
Source: PLoS One. 2012 Jun 26;7(6):e39456. doi: 10.1371/journal.pone.0039456 (PMC3383708; doi:10.1371/journal.pone.0039456)
Supplement: Table S2 — List of genes upregulated both in GCN5 KO NSC and in N-Myc KO NSC. Genes are listed by name and sorted in descending order by fold change in the GCN5 KO. (PDF) [file pone.0039456.s002.pdf]

Supplemental Table S2 Genes upregulated both in GCN5 and N-Myc KO NSC

TargetID	Expression KO/Control GCN5	Expression KO/Control N-Myc
PRSS12	2809.8	3.3
GSDM3	106.9	2.6
D630014E21RIK	104.4	3.4
9630017O17	75.0	2.4
IL1RL2	68.5	9.6
5430433G21RIK	42.3	4.1
PIK3C2G	34.7	9.9
MITF	34.5	32.2
SULT2A1	34.1	2.4
PYGM	32.7	88.6
AI316828	31.9	7.7
LOC386056	31.3	2.6
PTPRD	31.1	11.5
DBCCR1	27.8	505.8
NETO1	24.9	3.0
TRBV1	24.2	21.8
C230084J24RIK	23.8	3.2
4921504P13RIK	23.7	2.0
LOC329664	23.0	16.2
SCL0002180.1_25	20.1	7.4
LIN7A	19.7	17.1
A830016G23RIK	19.5	2.2
ROR2	19.4	7.2
2600013E07RIK	18.0	2.5
A130034M23RIK	16.9	3.5
2700089E24RIK	16.3	3.1
SLC6A4	16.2	48.3
VGLL2	16.0	640.3
VIL1	15.7	3.2
9630035P19RIK	15.5	2.1
SPEER4D	15.5	8.1
4933406L09RIK	14.7	6.3
CACNB4	14.6	2.3
A730081H18RIK	14.3	3.9
LIN7A	14.2	17.0
2300003P22RIK	13.4	3.2
C030034P18RIK	13.2	35.8
H2-T3	12.9	2.1
ADAM23	12.9	8.2
PACE4	12.0	3.4
SCL0002779.1_2	11.9	3.4
SLC11A1	11.8	56.6
LOC385139	11.7	3.3
ALDH7A1	11.6	6.5
OLFR453	11.3	4.7
POU2F2	11.3	2.7
AI593442	11.2	27.5
EFNA5	11.2	104.7
TNFSF6	10.7	3.3
LOC223672	10.6	3.3
SORCS3	10.6	139.5
COL11A1	10.4	9.4
5430431D22RIK	9.4	5.2
9630013P03RIK	9.3	2.0
AF067061	9.3	2.5
NCAML	9.0	2.9
4833446E11RIK	9.0	2.7
AFFX-BIOC-5-AT_231	8.9	2.4
AW046396	8.8	10.8
IFITM2	8.6	3.2
MRGPRA6	8.5	17.0
ANK3	8.3	2.5
CYP11A1	8.0	3.7
SCL000068.1_96_REVCOMP	8.0	7.0
ART3	7.9	3.5
4930553I04RIK	7.9	3.3
ITGBL1	7.8	3.6
9430064K01RIK	7.7	10.1
LOC328082	7.6	2.7
ADAMTS13	7.5	2.8
SLC6A6	7.5	2.6
FGD2	7.4	4.1
XKH	7.4	3.1
MYH14	7.2	4.4
C030016D13RIK	7.1	13.7
IFITM2	7.1	3.5
4931431F19RIK	6.9	2.8
EPPB9	6.9	44.1
SOD3	6.8	2.5
C130036K02	6.8	3.3
FIBCD1	6.8	2.9
LOC385626	6.7	19.9
4930415N18RIK	6.7	2.6
OLFR1134	6.7	3.0
EDA2R	6.7	2.0
4932430A15RIK	6.6	2.3
D130011M18RIK	6.5	5.2
SLITRK1	6.5	3.9
C730031G17	6.4	3.4
B3GAT3	6.2	3.1
C130057K09	6.2	2.8
E130115E03RIK	6.2	3.4
ASTN2	6.2	2.1
B3GALT5	6.0	9.5
KCNMA1	5.8	3.2
4921528I01RIK	5.8	4.1
PTPRO	5.8	64.4
9530020O07RIK	5.8	3.7
C230053B09RIK	5.8	3.9
1700108N11RIK	5.5	2.5
BC034076	5.5	3.1
C130012C08RIK	5.4	4.3
LOC383516	5.4	4.3
2900076G11RIK	5.4	2.2
4921511H13RIK	5.3	37.5
1700019E08RIK	5.3	2.2
1700110N18RIK	5.3	3.9

Supplemental Table S2 Genes upregulated both in GCN5 and N-Myc KO NSC

NFATC1	5.2	3.2
GMPR	5.2	4.8
DMN	5.2	2.9
LOC381904	5.2	82.4
LOC383934	5.1	4.3
SYT1	5.1	104.1
CAPN5	5.0	11.0
RSPO3	5.0	470.8
COX8B	4.9	7.1
GBX2	4.8	5.2
SEMA6D	4.8	8.2
2900041A09RIK	4.7	2.2
LOC384376	4.7	2.9
PSPC1	4.6	3.5
MATP	4.6	12.1
C030011O14RIK	4.6	33.1
2900076A13RIK	4.5	3.8
D130049J17RIK	4.5	2.8
SCN7A	4.5	12.1
LOC242088	4.4	2.1
C230004H03RIK	4.4	6.2
C030011O14RIK	4.4	9.4
1700112L15RIK	4.4	2.7
DNAJC6	4.3	2.2
KLK21	4.3	3.0
3200001K10RIK	4.2	2.8
9130213B05RIK	4.2	7.6
CNTN1	4.2	2.0
D230040J21RIK	4.2	3.9
PDE8A	4.2	6.6
CDKN1A	4.2	2.4
5730435O14RIK	4.2	39.9
SULT1A1	4.2	2.1
D18ERTD232E	4.2	8.1
E030040J22RIK	4.2	2.2
9030624G23RIK	4.1	3.3
GALNTL4	4.1	2.3
4930544G21RIK	4.1	10.7
LOC382229	4.1	181.8
RELN	4.0	2.3
LALBA	4.0	3.7
MFI2	4.0	2.2
ABI3	4.0	5.6
C130069I09	3.9	2.6
B130050K08	3.9	2.3
SORCS2-PENDING	3.9	3.8
DIRAS2	3.9	34.2
HELB	3.9	5.6
GYPA	3.8	10.4
6720431C02RIK	3.8	11.0
CBFA2T3H	3.8	6.2
LOC382386	3.8	2.8
RPTN	3.8	2.2
A030013D21	3.8	4.6
MTAP4	3.7	6.9
SORCS1	3.7	3.3
D630040I23RIK	3.7	2.4
2210012C09RIK	3.6	2.1
LOC384345	3.6	3.3
2310079P10RIK	3.6	28.1
6530405K19	3.6	2.6
COL28A1	3.6	9.4
CDKN1A	3.6	2.1
PTGES	3.5	2.1
LOC381185	3.5	2.2
CDH6	3.5	6.0
A2BP1	3.5	17.8
POU2F1	3.5	2.2
PCDHGB2	3.5	10.8
5430439G13RIK	3.5	2.5
ADAM23	3.5	7.1
1110001P11RIK	3.5	8.8
G2-PENDING	3.5	16.7
APS-PENDING	3.5	4.1
9530029F08RIK	3.5	4.6
LGI2	3.5	14.1
LOC331028	3.4	4.3
LOC385163	3.4	2.4
TNFSF11	3.4	2.7
PTPRK	3.4	9.4
GGN	3.4	4.4
APBB1	3.4	24.4
GAB2	3.4	7.5
A130004G07RIK	3.3	2.8
4930403D09RIK	3.3	3.1
SCARB1	3.3	2.8
PTPRD	3.3	10.2
ZMYND17	3.2	51.5
6430524E21RIK	3.2	2.5
CNTN1	3.2	2.2
TNC	3.2	4.1
CHRNA2	3.2	2.1
LIN7A	3.2	9.0
2310061A09RIK	3.2	2.9
DUSP4	3.2	10.0
9130214F15RIK	3.1	4.9
NDUFA6	3.1	4.1
9230116M18RIK	3.1	2.4
VAMP1	3.1	2.7
9430095B17RIK	3.1	26.7
SCL000843.1_27	3.1	3.6
POU4F3	3.1	2.9
SLCO3A1	3.1	4.6
NELL2	3.1	2.8
HRC	3.1	7.5
BRI3BP	3.1	3.5
AW121567	3.1	10.1
LOC386275	3.1	2.5
CNKSR2	3.1	6.0
ZFP26	3.0	22.1

Supplemental Table S2 Genes upregulated both in GCN5 and N-Myc KO NSC

2300002D11RIK	3.0	4.9	SIAT7E	2.6	6.6
A830039N20RIK	3.0	5.2	FREQ	2.6	3.0
CEBPA	3.0	2.5	1700110N18RIK	2.6	3.1
LOC384433	3.0	2.3	HOXB13	2.6	2.4
NELL2	3.0	3.1	LOC381758	2.6	3.1
SH2D2A	3.0	2.1	SV2A	2.6	199.8
5330416C01RIK	3.0	3.6	OLFR1330	2.6	2.5
KCNMA1	3.0	2.1	PTPRK	2.6	3.6
8030462N17RIK	3.0	4.4	NEGR1	2.6	2.3
AK122525	2.9	3.8	GPR49	2.6	3.4
SLC5A9	2.9	2.6	DSC1	2.5	3.4
LOC381736	2.9	2.7	1700023G09RIK	2.5	9.6
NELL2	2.9	2.1	KSR	2.5	150.9
SORCS2	2.9	2.5	1700085N21RIK	2.5	71.9
FLRT3	2.9	3.3	AW125391	2.5	2.6
4930564B12RIK	2.9	2.4	TRIM33	2.5	3.3
AI505012	2.9	4.6	IGF2R	2.5	4.5
LMCD1	2.9	14.4	SORCS1	2.5	4.6
SLC24A3	2.9	6.4	ABCA9	2.5	2.8
NGFR	2.9	2.2	HSD11B1	2.5	10.8
A330058M13RIK	2.9	9.1	COMTD1	2.5	3.3
IGF2BP1	2.9	2.4	FZD4	2.5	3.1
A230063O03RIK	2.9	2.4	SLCO3A1	2.5	6.9
CEBPA	2.9	7.1	DUSP4	2.5	5.3
BVES	2.9	9.5	NETO1	2.5	3.1
B130017P16RIK	2.8	5.8	TNC	2.5	2.9
FGF1	2.8	5.1	MARK1	2.5	2.6
D430039N05RIK	2.8	2.3	2900072G11RIK	2.5	3.3
5930405F01RIK	2.8	2.2	4833424O15RIK	2.5	2.6
6430594K11RIK	2.8	2.1	SCRN1	2.5	3.4
E130310K16RIK	2.8	2.4	D330050I23RIK	2.5	2.4
CACNA2D3	2.8	3.0	ADAM12	2.5	5.9
LMO2	2.8	14.2	MKIAA1457	2.5	2.3
TNC	2.8	3.9	PTPNS1	2.4	2.7
6330540D07RIK	2.8	4.8	ENO2	2.4	3.1
4930577M16RIK	2.7	7.4	PLXNA4	2.4	2.2
4933412E14RIK	2.7	3.3	ZFP532	2.4	2.4
B230308G19RIK	2.7	6.3	DSCAM	2.4	2.1
JUB	2.7	7.1	FGF1	2.4	10.6
CDKN2B	2.7	14.0	LOC381650	2.4	3.0
EHD4	2.7	2.8	LOC381142	2.4	4.3
5730507A09RIK	2.7	7.9	GPC6	2.4	2.8
A430102L10	2.7	8.7	NANOS1	2.4	3.3
AI429613	2.7	3.5	PTPNS1	2.4	12.2
ELMO1	2.7	3.5	VEGFC	2.4	2.3
B130021B11RIK	2.7	6.6	2810022L02RIK	2.4	2.5
A230092L08RIK	2.7	13.4	HIP1	2.4	2.6
NSD1	2.7	3.4	A130082F03RIK	2.4	6.0
4930488B01RIK	2.7	2.6	ATP8A1	2.4	2.4
PSCDBP	2.6	4.5	OLFR262	2.4	26.1
1700128F08RIK	2.6	2.8	PEM	2.4	2.2
CACNA1G	2.6	4.6	SRMS	2.4	2.2
OLFR1402	2.6	2.2	PDGFC	2.3	2.8
ABCA5	2.6	9.6	PAQR6	2.3	2.1
4930473H19RIK	2.6	2.8	PIRA5	2.3	3.0

Supplemental Table S2 Genes upregulated both in GCN5 and N-Myc KO NSC

4930458B22RIK	2.3	25.4
IGHV1S122_AF02	2.3	2.0
LOC193403	2.3	4.6
BC037006	2.3	2.4
COL5A1	2.3	7.6
GPC6	2.3	5.5
LOC240023	2.3	26.8
GM644	2.3	1742.5
4833446K15RIK	2.3	2.8
KCTD12	2.3	3.0
ASS1	2.3	6.4
AI504432	2.3	6.6
LOC381749	2.3	17.0
THEG	2.3	2.9
ADAMTS1	2.3	5.5
SLCO3A1	2.3	4.9
ZFP612	2.3	10.3
SYT9	2.3	5.5
D030011O10RIK	2.3	2.1
LOC242004	2.3	3.4
LOC381351	2.3	2.5
DUSP15	2.3	5.6
TMC5	2.3	3.4
NFAT5	2.3	10.6
RYR2	2.3	40.8
IGFBP7	2.3	7.0
BC003277	2.3	3.8
SIAT7D	2.3	13.1
CHRM3	2.3	13.8
LOC268958	2.2	2.2
LOC382017	2.2	19.7
KNSL1	2.2	5.7
LOC226990	2.2	7.6
4933404M02RIK	2.2	2.9
SULT1A1	2.2	4.0
FLRT3	2.2	9.3
CYP2B19	2.2	2.2
C130010K08RIK	2.2	2.4
SV2A	2.2	4.7
SDPR	2.2	2.7
SCL0002785.1_49	2.2	2.4
LOC245510	2.2	2.2
9430046I01RIK	2.2	3.3
MISC12	2.2	3.7
ESAM1	2.2	5.3
B3GALT4	2.2	7.3
D030072B18RIK	2.2	4.0
1700008A07RIK	2.2	3.2
SCL00104366.1_6	2.2	2.2
HOXA2	2.2	4.6
BC022224	2.2	6.3
D630004K10RIK	2.2	5.6
EFNB2	2.2	102.9
REPRIMO	2.2	5.9
CACNA2D2	2.2	2.1
SERPINE2	2.2	3.6
B230362M20RIK	2.2	7.4
C130007D14	2.2	2.9
4933417N07RIK	2.2	2.5
ADAMTS5	2.2	4.9
2700004G04RIK	2.1	2.1
KCTD12	2.1	2.2
LOC279056	2.1	12.9
EIF5A2	2.1	4.2
STK3	2.1	2.1
4833424O15RIK	2.1	2.3
D630002J18RIK	2.1	3.2
RIMS4	2.1	2.8
SEMA6D	2.1	2.1
TXK	2.1	2.4
SAMD11	2.1	11.1
OMP	2.1	10.6
AI854635	2.1	11.0
C030034I22RIK	2.1	5.0
TGFBR3	2.1	4.4
RAPGEF5	2.1	2.4
9230106D23RIK	2.1	2.1
NINJ1	2.1	2.7
KLRI1	2.1	6.3
EIF2S1	2.1	6.7
BMPER	2.1	2.3
RNPC1	2.1	2.5
COL11A1	2.1	6.5
EGFL7	2.1	3.3
4930520C08RIK	2.1	9.7
4930565A21RIK	2.1	2.1
SPRY4	2.1	7.1
5730409G07RIK	2.1	2.5
SERPINF1	2.1	7.5
D630011N09RIK	2.1	8.4
LOC381683	2.1	2.1
SCL0001284.1_18	2.1	228.5
5033415L01RIK	2.1	5.5
C330023J09RIK	2.1	2.6
RLN1	2.1	2.7
PECAM1	2.0	5.6
2900046G09RIK	2.0	6.5
GABRA2	2.0	4.2
4933400F03RIK	2.0	4.0
SCL0002855.1_1056	2.0	2.8
4930506K12RIK	2.0	7.8
6430530L21RIK	2.0	2.9
1700109G14RIK	2.0	5.8
A030007L22	2.0	2.6
HIST1H2AC	2.0	3.5
1110033L15RIK	2.0	2.7
CSMD1	2.0	3.5
PDGFC	2.0	3.0
1700010K23RIK	2.0	2.4
C630017J20RIK	2.0	2.5

Supplemental Table S2 Genes upregulated both in GCN5 and N-Myc KO NSC

B830020B14RIK	2.0	2.3
BC060737	2.0	3.2
DPP6	2.0	17.9
AK4	2.0	2.6
MSI1H	2.0	3.1
